# Sorbent-coated metal discs for time-integrated VOC sampling: A reproducible workflow coupled to SPME–GC/MS

**DOI:** 10.1016/j.mex.2026.104015

**Published:** 2026-06-25

**Authors:** Robert Lundberg, Thomas Lundeberg, Johan Lundeberg, Johan Dahlén

**Affiliations:** a Chromalytica AB, Ullared, SE-311 61, Sweden; b Department of Physics, Chemistry and Biology, Linköping University, Linköping, SE-581 83, Sweden; c Department of Physiology and Pharmacology, Karolinska Institutet and Sabbatsbergssjukhus, Stockholm, SE-182 57, Sweden; d Department of Clinical Physiology, Skanes universitetssjukhus Lund, Lund, Skåne, Sweden

**Keywords:** Carbopack X, VOC, Breath analysis, GC/MS, Time-integrated sampling, Re-adsorption, Headspace

## Abstract

We describe a time-integrated platform based on sorbent-coated metal discs, formalized as a configurable architecture for VOC enrichment prior to gas chromatography/mass spectrometry (GC/MS) via secondary re-adsorption onto a solid-phase microextraction (SPME) fiber. A reproducible fabrication, conditioning, sampling, desorption, and re-adsorption protocol is specified, with a sorbent loading of approximately 0.2 g per 3.6 cm disc and sampling durations of 60 min. Across standard analysis replicates, the disc workflow produced higher GC/MS peak areas than direct SPME for most of the analytes, with reduced response for highly volatile compounds such as acetone. To explain this volatility-dependent behavior, we introduce a mass–transport competition framework in which the measurable signal is governed by the product of accumulated analyte mass and transfer efficiency, S = M_ads × η_transfer. The framework distinguishes the capacity-driven platform from equilibrium-based SPME. The described workflow is solvent-free, low-waste, reusable, and compatible with routine GC/MS instrumentation. Method overview:

• Sorbent discs are prepared by covering a metal plate with a layer of sorbent material using a silicon adhesive.
• Conditioned sorbent discs can be exposed to any gaseous samples.
• Sorbent discs are heated to desorb the enriched VOCs that are subsequently re-adsorbed onto an SPME fiber to enable GC/MS analysis.

## Graphical abstract

**Figure:**
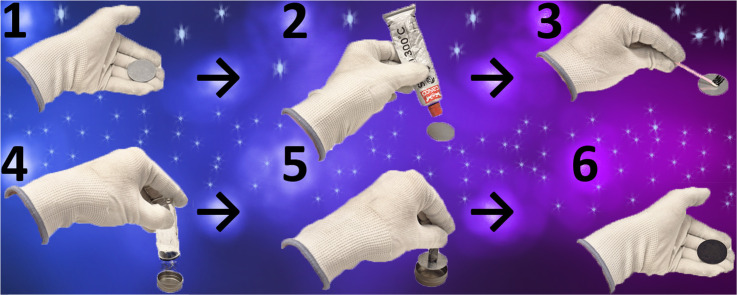

## Specifications table

**Table:** 

**Subject area**	Chemistry
**More specific subject area**	Analytical chemistry
**Name of your method**	Sorbent-coated metal disc workflow for time-integrated VOC sampling
**Name and reference of original method**	Not applicable
**Resource availability**	Agilent 6890 GC instrument equipped with an DB-5MS UI ultra inert 5% phenyl, 95% methyl (30 m x 0.25 mm x 0.25 µm) capillary column (cat. no. 122–5532UI) and a 5973 Mass Selective Detector; Merck CAR/PDMS, 24 ga, 85 μm SPME fiber (cat. no. 57,334-U); Merck 60–80 mesh, Carbopack™ X, sorbent (cat. no. 10,436); heat-resistant silicone adhesive Casco silikon 300 °C

## Background

Analysis of volatile organic compounds (VOCs) in gaseous matrices, including exhaled breath, ambient air, and enclosed headspace systems, is widely applied in analytical chemistry, environmental monitoring, and biomedical research [[1], [2], [3]]. Gas chromatography–mass spectrometry (GC/MS), often combined with solid-phase microextraction (SPME), remains a standard analytical platform for VOC detection due to its sensitivity and compatibility with complex mixtures [4,5]. In many established workflows VOCs are collected using short-duration ("snapshot") sampling strategies; bag-based collection followed by extraction, or brief direct exposure of an SPME fiber [4,6]. These approaches are practical and widely used but primarily capture instantaneous concentrations, which may not represent time-averaged exposure when emissions fluctuate due to dynamic sources, environmental conditions, or physiological processes [6,7].

Time-integrated sampling provides a complementary approach by allowing analytes to accumulate over extended periods, reflecting cumulative exposure rather than instantaneous concentration [6]. Sorbent-based techniques are well suited for time-integrated sampling, but commonly used formats such as SPME fibers and sorbent tubes are typically constrained either by limited sorbent capacity (SPME) or by reliance on controlled sampling geometry and volumetric flow (sorbent tubes). These constraints can affect reproducibility and comparability across studies, particularly under non-standardized or field conditions [4,6].

A practical methodological challenge is therefore to achieve high-capacity time-integrated VOC capture under conditions where sampling geometry and volumetric flow are not strictly controlled, while maintaining compatibility with standard analytical platforms and ensuring reproducibility of the workflow. The present work addresses this challenge by introducing a sorbent-coated metal disc workflow in which a high-capacity granular sorbent (Carbopack X) is immobilized on a stainless-steel substrate with a heat-resistant silicone adhesive. The disc is exposed directly to the sampling environment, allowing analytes to diffuse and adsorb onto the sorbent layer. Following sampling, analytes are thermally desorbed in a sealed container, and the resulting headspace is transferred to a CAR/PDMS SPME fiber for direct compatibility with standard split/splitless GC/MS inlets.

The contribution of this paper is a fully specified, reproducible, and transferable implementation protocol for the sorbent-coated disc workflow, including disc preparation, conditioning, sampling, thermal desorption, and analytical transfer, together with operational parameters, reproducibility and validation data supporting practical implementation. The focus is on methodological clarity and reproducibility rather than on application-specific performance. A companion paper [2] reports the application of this workflow to exhaled-breath sampling for early lung cancer screening. The aim of that study was application. The aim of the present manuscript is different: to formalize the disc as a generalizable analytical platform and to provide the methodological information required for transfer to other laboratories and matrices. The main contributions of the present work are an introduction of a mass–transport competition framework, S = M_ads × η_transfer, that describes signal formation in this capacity-driven, non-equilibrium regime and predicts the volatility-dependent behavior observed for highly volatile analytes, a demonstration that both the sorbent and the substrate are interchangeable without loss of function, establishing the disc as a configurable architecture rather than a single device, and a specification of the reproducibility envelope, blank-control strategy, and green-chemistry profile required for inter-laboratory transfer.

## Method details

### Overview of workflow

**Fig. 1:**
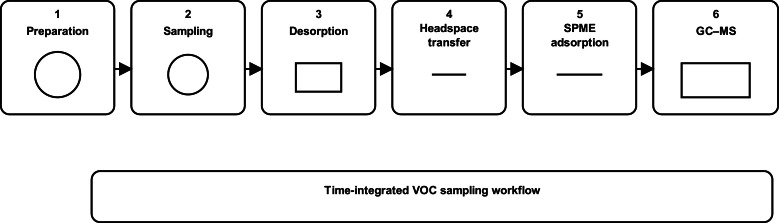
Schematic representation of the sorbent disc workflow: (1) preparation of sorbent-coated disc, (2) exposure to gaseous sample, (3) thermal desorption in sealed container, (4) headspace collection, (5) re-adsorption onto SPME fiber, and (6) GC–MS analysis. The workflow illustrates the full sequence required for reproducible time-integrated VOC sampling and analysis.

A schematic overview of the workflow is shown in Fig. 1. The workflow consists of six sequential steps: (1) preparation of the sorbent-coated disc, (2) passive sampling of the gaseous matrix, (3) thermal desorption in a sealed container, (4) headspace collection into a glass syringe, (5) re-adsorption of the headspace VOCs onto a CAR/PDMS SPME fiber, and (6) GC/MS analysis.

### Materials

The bill of materials is given in Table 1. The prototype implementation used: stainless-steel discs (3.6 cm diameter, 0.5–1.0 mm thickness); Carbopack X sorbent (60–80 mesh; Merck); a heat-resistant silicone adhesive (Silikon 300 °C, Casco) [8]; high-purity nitrogen for conditioning; CAR/PDMS SPME fiber (85 µm; Merck) [9]; a sealed metal desorption chamber (∼15 mL internal volume) fitted with a gas-tight septum; a gas-tight glass syringe (10–20 mL); pre-punched rubber stoppers for syringe sealing; nitrile gloves and tweezers for handling. The estimated consumable cost per disc is below €5, excluding reusable equipment such as the GC/MS instrument and laboratory oven.

**Table 1:** Bill of materials, with a total consumable cost per disc: < €5.

Item	Component	Specification	Approx. cost (€)
1	Metal disc substrate	Stainless steel, Ø 3.6 cm, 0.5–1.0 mm	0.5–1.0
2	Sorbent material	Carbopack X, 60–80 mesh (∼0.2 g per disc)	1.0–1.5
3	Adhesive	Heat-resistant silicone (rated ∼300 °C)	0.2–0.4
4	Conditioning gas	High-purity nitrogen	<0.1
5	Conditioning container	Sealed metal container with N₂ port	reusable
6	Desorption chamber	Sealed metal container, ∼15 mL, septum-equipped lid	0.5–1.0
7	Septa	GC injection septa, gas-tight	0.2
8	Glass syringe	10–20 mL gas-tight	reusable
9	Rubber stopper	Pre-punched	0.1
10	Secondary sampler	CAR/PDMS SPME fiber, 85 µm	reusable

### Sorbent disc preparation

Stainless-steel discs were rinsed with methanol, air-dried, and handled with gloves and tweezers. A thin, uniform layer of silicone adhesive was applied to one face of the disc using a cotton swab. The adhesive-coated face was immediately brought into contact with Carbopack X powder by pressing the disc into the powder; excess sorbent was removed by gentle tapping. The mass of sorbent deposited was determined gravimetrically. The target sorbent mass per disc was approximately 0.2 g. After coating, discs were thermally cured under nitrogen at 280 °C for 40 min with a flow rate of approximately 40 mL min⁻¹ in a sealed metal container. The cured discs were inspected by eye and by optical microscopy to confirm uniform sorbent coverage.

### Routine conditioning

Before each sampling experiment, discs were re-conditioned at 250 °C for 30 min under a nitrogen flow of 40 mL min⁻¹ in a sealed metal container. *Re*-conditioning removes residual contaminants accumulated during storage and stabilizes the background signal prior to sampling.

### Sampling procedure

Sampling is based on direct exposure of the conditioned disc to the gaseous matrix, allowing time-integrated accumulation of analytes. Typical exposure times were 60 min as previously justified [1], although shorter exposure times such as 45 min were also used. The sampling configuration depends on the matrix: breath sampling was performed by positioning the disc approximately 1 cm from the mouth using a holder; ambient sampling was performed by exposing the disc directly in the environment without enclosure; headspace sampling was performed by suspending or placing the disc inside a sealed container. For reliable comparison within an experiment, the sampling geometry must be kept consistent and the exposure time must be recorded precisely.

### Thermal desorption and headspace transfer

After sampling, the disc was placed inside the sealed desorption chamber (∼15 mL), the chamber was sealed with a septum-equipped lid, and the chamber was heated to 250 °C for 6 min. Immediately after heating, the headspace (∼15 mL) was withdrawn into a gas-tight glass syringe through the septum. Delay between heating and headspace collection was minimized to reduce loss of volatile analytes.

### SPME re-adsorption

The syringe containing the desorbed headspace was sealed with a pre-punched rubber stopper. The CAR/PDMS SPME fiber [9] was inserted through the stopper and exposed to the headspace inside the syringe for 35 min, allowing the analytes to re-adsorb onto the fiber coating. The two-step transfer (disc desorption → syringe headspace → SPME fiber) concentrates the analytes onto a standard SPME fiber, enabling direct GC/MS analysis through a conventional split/splitless inlet without specialized desorption interfaces.

### GC/MS analysis

After re-adsorption, the SPME fiber was inserted directly into the injection port of the GC/MS system. Samples were injected splitless to maximize analyte transfer. The GC/MS configuration used in the prototype implementation was an Agilent 6890 gas chromatograph coupled to a mass-selective detector operated in scan mode (*m/z* 30–350, ∼2.3 scans s⁻¹). Column type, carrier gas, and temperature program were selected according to the analytical objective and are not expected to influence the general applicability of the workflow. Data analysis was performed using ChemStation software with NIST spectral libraries for compound identification.

### Signal behavior theory

**Fig. 2:**
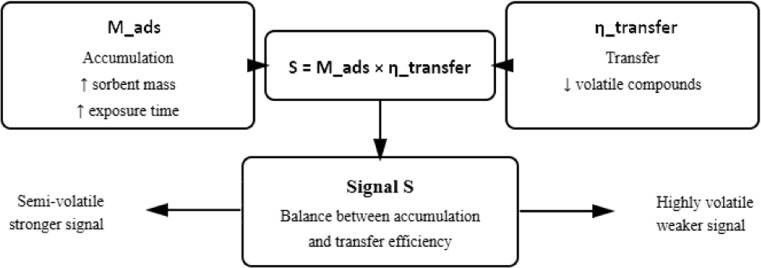
Schematic representation of signal formation in the sorbent disc workflow. The measured signal (S) is determined by the balance between accumulated analyte mass (M_ads), which increases with sorbent capacity and exposure time, and transfer efficiency (η_transfer), which may decrease for highly volatile compounds. This model provides a practical interpretation of compound-dependent signal behavior observed under the tested conditions.

This behavior can be interpreted using a simplified framework in which the measured signal (S) reflects the combined effects of accumulated analyte mass (M_ads) and transfer efficiency (η_transfer), as illustrated in Fig. 2:

$$S=M_{\text{ads}}\times \eta_{\text{transfer}}$$

In this framework, transfer efficiency reflects how efficient the conversion from captured analyte mass to detected analytical signal is. Increased exposure time promotes analyte accumulation, while transfer efficiency may vary depending on compound properties and handling conditions. These observations are consistent with the conceptual framework of signal formation described in Fig. 2, in which compound-dependent signal behavior reflects the balance between analyte accumulation and transfer efficiency.

## Results

This section reports the validation experiments performed to support practical implementation. The experiments are designed to demonstrate workflow reproducibility, characterize key sources of variability, and document the signal behavior under representative conditions. They are not intended as a comprehensive analytical validation; absolute quantification, matrix-effect characterization, and inter-laboratory transferability are out of scope for this work and are listed as future work.

### Surface characterization and adsorbent properties

**Fig. 3:**
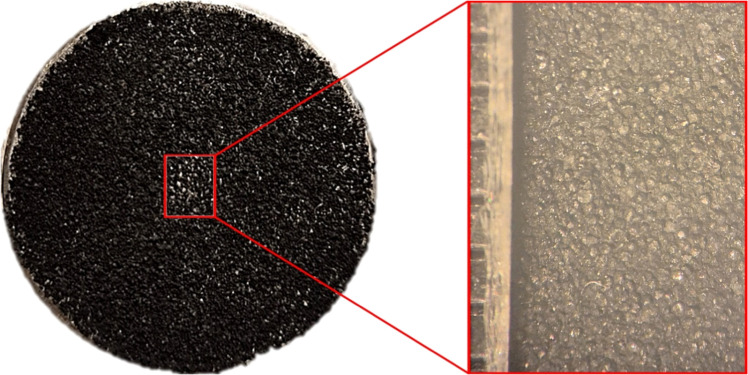
Photograph (left) and optical microscopy image (right) of a prepared sorbent-coated metal disc. The images demonstrate uniform distribution of Carbopack X on the adhesive-coated stainless-steel substrate. The visible granularity reflects the 60–80 mesh sorbent structure.

The performance of the sorbent disc platform depends on the physicochemical character of the immobilized sorbent layer, the accessibility of the sorbent surface to the gas phase, and the stability of the surface across the conditioning and desorption cycles. Optical microscopy at 5× magnification (Fig. 3) confirms a uniform, contiguous distribution of the granular sorbent across the metal disc surface, with no visible bare patches or aggregated clumps. The sorbent loading determined gravimetrically averages 0.2 g per 3.6 cm disc.

For the primary sorbent used in this work, Carbopack X, the surface and adsorptive properties have been extensively characterized in the engineered-carbon-adsorbent literature [10]. Carbopack X is a graphitized carbon black with a specific surface area of approximately 240 m²/g, a predominantly mesoporous rather than microporous structure, and a graphene-like sp² carbon surface that is essentially nonpolar and strongly hydrophobic. Each of these properties is relevant to the sampling regime described here. The high specific surface area provides the adsorptive capacity that the M_ads term in the mass–transport competition framework requires for time-integrated enrichment of semi-volatile analytes. The mesoporous rather than microporous structure allows reversible thermal desorption without the irreversible-binding and slow-release kinetics that microporous activated carbons exhibit, which is what makes the two-step desorption-and-re-adsorption transfer to the SPME fiber practical. The hydrophobic graphitic surface limits competitive adsorption of water from exhaled breath or humid ambient air, a recognized confounder in VOC analysis of biological matrices and one that more polar adsorbents (oxidized activated carbons, ordered mesoporous carbons with polar surface groups) do not control as effectively [10].

The thermal stability of high-temperature silicone adhesives at the temperatures used in the present workflow (initial cure at 280 °C for 40 min, routine conditioning at 250 °C for 30 min) is supported by an established body of literature on polysiloxane thermal degradation pathways and on the outgassing behavior of cured silicone elastomers [8,11]. The principal sources of silicone-derived background contamination in GC/MS analysis are residual cyclic siloxane oligomers, predominantly hexamethylcyclotrisiloxane (D3, *m/z* 207), octamethylcyclotetrasiloxane (D4, *m/z* 281), decamethylcyclopentasiloxane (D5, *m/z* 355), and dodecamethylcyclohexasiloxane (D6, *m/z* 429). These siloxanes are formed during silicone curing and volatilize progressively as the temperature increases [12]. The 280 °C initial-cure step in the present preparation protocol exceeds the volatilization thresholds of D3 through D5 cyclic siloxanes and substantially depletes the residual cyclic-oligomer pool before the disc enters analytical use; the routine 250 °C conditioning before each sampling refreshes this depletion. This rationale, combined with the conditioned-disc blank chromatogram, in the "Background and blank control" section, showing no detectable D3–D6 peaks at the retention windows of the target analytes, addresses the silicone-adhesive interference concern at both mechanistic and empirical levels. Detailed characterization of the cured adhesive after repeated thermal cycling is identified as an additional analytical input in the future development pathway.

**Fig. 4:**
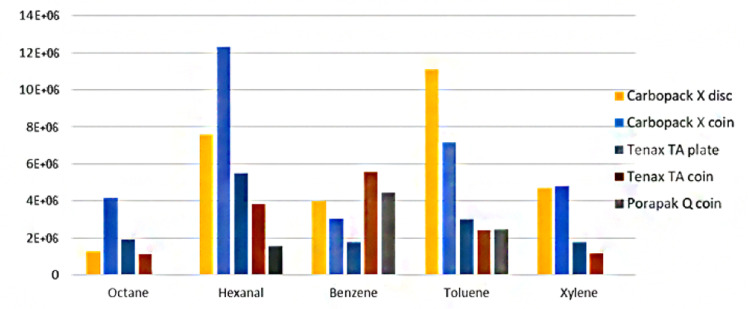
GC/MS (6890) peak areas (n = 1) of five key compounds after 60 min of breath sampling using five types of sorbent discs: a carbopack X sorbent disc as described in the method details section (yellow), a carbopack X sorbent disc prepared using an old 28.5 mm diameter Swedish five kronor coin (of the type issued between 1976 and 1992) (light blue), a rectangular metal plate covered with Tenax TA sorbent powder (dark blue), an old 28.5 mm diameter Swedish five kronor coin (of the type issued between 1976 and 1992) covered with Tenax TA sorbent powder (brown), and an old 28.5 mm diameter Swedish five kronor coin (of the type issued between 1976 and 1992) covered with Porapak Q sorbent powder (grey). Exhaled breath from the same subject (the corresponding author) was sampled using the five discs, with each disc used at a different time point. Carbopack X sampling produced the highest overall values, while the other sorbent materials each showed distinct advantages. Surprisingly, the coin-based sampling materials also performed well.

**Fig. 5:**
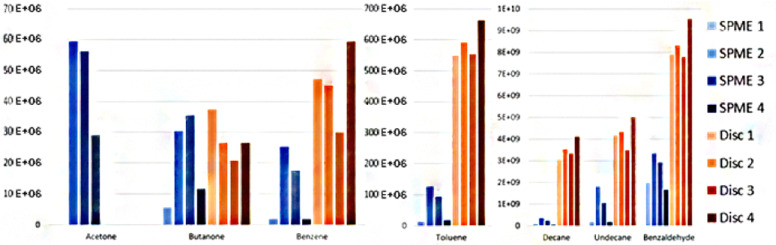
GC/MS standard (20 ppm) analysis peak areas (n = 4) using two sampling methods: SPME-GC/MS (blue) and DISC-SPME-GC/MS (orange brown), both with a 45-minute sampling time. All compounds except the earliest eluting compound, acetone, exhibit higher peak area values for the sorbent disc method compared to direct SPME extraction.

The platform also tolerates substitution to the two polymeric sorbents used in the configurability demonstration in Fig. 4: Tenax TA, a poly(2,6-diphenyl-p-phenylene oxide) resin with a specific surface area of approximately 35 m²/g and a hydrophobic aromatic surface, and Porapak Q, an ethylvinylbenzene–divinylbenzene copolymer with a specific surface area of approximately 550–600 m²/g and a more polar surface chemistry. These polymeric sorbents differ from Carbopack X in surface area, surface chemistry, thermal stability, and adsorption mechanism (predominant gas-solid partition into the polymer matrix rather than gas-solid adsorption on a graphitic surface), and they yield correspondingly different absolute peak-area responses for the compounds tested in Fig. 4 and Fig. 5. The architecture-level result, however, is that the disc platform is not contingent on one specific surface chemistry; discs prepared with each of these three sorbents on each of three different metal substrates produce functional, signal-yielding samplers. The mass–transport competition framework introduced in Fig. 2 applies to all three sorbents, with the partitioning of signal between M_ads and η_transfer terms shifting according to the surface and porous-structure properties of the chosen sorbent and the volatility of the analyte.

### Sorbent disc performance across sorbents and substrates

The sorbent disc method is highly versatile, as discs can be prepared using any thin metal plate, heat-resistant adhesive, and sorbent powder. To demonstrate this, five different types of sorbent discs were prepared according to the method section. The five discs were used to sample exhaled breath, using 60-minute sampling times according to the method section, followed by analysis of the collected samples using the described 6890 GC instrument, yielding the data behind Fig. 4. Exhaled breath from the same subject (the corresponding author) was sampled using the five discs, with each disc used at a different time point. Fig. 4 shows exhaled breath peak areas (n = 1) for five VOC samples collected with discs made from different metal plates and adsorbents: yellow bars represent the standard Carbopack X sorbent disc, light blue bars a disc prepared from an old coin with Carbopack X, dark blue bars a rectangular plate coated with Tenax TA, brown bars an old coin coated with Tenax TA, and grey bars an old coin coated with Porapak Q. Further details are provided in the figure caption.

The yellow and light blue bars in Fig. 4 indicate that Carbopack X is more suitable for the extraction of VOCs from exhaled breath than the sorbent materials Tenax TA and Porapak Q. The light blue bars in Fig. 4 illustrate how well the sorbent disc concept works even when an old coin is used as the base.

### Minimal validation and method comparison using standard analysis

To support practical implementation of the sorbent disc workflow, a few replicates of standard mixture analyses were carried out for a controlled method comparison. A standard solution of seven compounds dissolved in methanol at a concentration of 20 ppm was transferred (0.5 mL) to a 1-L glass container and Carbopack X discs were positioned in each container to sample the analytes from the headspace. The glass containers were sealed with aluminum foil that was penetrated with SPME-needles for simultaneous sampling of the standard mixture using both SPME and sorbent discs. The resulting measurements are presented as bar charts in Fig. 5. This figure shows average peak area values for sampling with SPME (SPME-GC/MS; n = 4) and sorbent discs (DISC-SPME-GC/MS; n = 4). The standard solution contained acetone, butanone, benzene, toluene, decane, undecane, and benzaldehyde.

The standard analysis results in Fig. 5, indicate that the sorbent discs sampled all compounds more effectively than SPME alone, except for the earliest eluting compound, acetone.

### Background and blank control

**Fig. 6:**
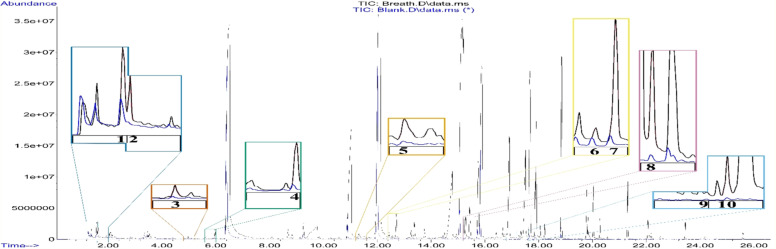
Overlay of total ion chromatograms, each normalized by their highest peak, from a breath sample (black) and a room air blank (blue) sampled using the sorbent discs. Peaks belonging to acetone (1), isoprene (2), toluene (3), hexanal (4), benzaldehyde (5), decane (6), octanal (7), nonanal (8), dodecane (9), and decanal (10) are enlarged. The larger black peaks indicate that the analytes originate from the exhaled breath.

It is important to perform routine blank analysis to identify background contributions and to monitor potential sources of contamination. Blank signals should be evaluated alongside sample measurements to ensure that analyte signals exceed background levels under the selected workflow conditions. Fig. 6 shows an overlay of TIC chromatograms from a room air blank analysis and breath sample analysis using sorbent discs to demonstrate that the observed signals originate from the sampled breath rather than the ambient air. The breath sample was taken from the corresponding author. The two total ion chromatograms compared are normalized together after the largest peak across both chromatograms, giving it an abundance of 100% and scaling everything else in both chromatograms down accordingly.

As can be seen in the chromatograms in Fig. 6, the peaks of target analytes (acetone, isoprene, toluene, hexanal, benzaldehyde, decane, octanal, nonanal, dodecane, and decanal) exhibit higher intensities in the breath sample as compared to the ambient air sample (blue). The presence of these compounds in the ambient air sample, although at lower concentrations, is logical given that ambient air inevitably contains traces of exhaled breath. Blank chromatograms demonstrated minimal siloxane-related interference within the analytical window of interest, confirming that adhesive-derived artifacts do not significantly impact VOC detection under the applied conditions. Blank analysis and evaluation were only done using the Carbopack X disc. However, because Tenax TA and Porapak Q are advantageous for extracting certain VOCs and can thus be relevant for other applications, blank analysis and evaluation of these sorbents represent an important future direction.

### Reproducibility and operational stability

Sorbent loading is controlled gravimetrically against a target of approximately 0.2 g per 3.6 cm disc. Across the independently prepared discs used in the experiments reported here, observed loadings spanned approximately 160–260 mg, with discs in this range producing qualitatively comparable analytical responses in the standard-mixture and breath-sampling experiments shown previously [2]. A formal statistical characterization of the loading distribution and a quantitative test of loading-vs-signal independence were outside the scope of the present platform-level description and are identified as future analytical inputs. Critical parameters requiring careful control to ensure reproducible results of the overall method have been identified. Table 2 provides an overview of the complete GC–MS workflow, summarizing each individual step, its specific purpose within the analytical process, and the corresponding critical control points that must be maintained to ensure reliable and reproducible results.

**Table 2:** Stepwise summary of the workflow, including purpose and critical control points for each stage.

Step	Description	Purpose	Critical control point
Disc preparation	Coating of metal disc with sorbent	Create adsorption surface	Uniform adhesive and sorbent distribution
Conditioning	Heating under inert gas	Remove contaminants	Temperature and duration control
Sampling	Exposure to gaseous sample	Accumulate analytes	Consistent geometry and exposure time
Thermal desorption	Heating in sealed container	Release analytes to gas phase	Sealing and timing
Headspace transfer	Gas withdrawal into syringe	Preserve analyte mixture	Rapid transfer after heating
SPME re-adsorption	Fiber exposure in headspace	Enable GC–MS compatibility	Exposure time consistency
GC–MS analysis	Instrumental detection	Identify analytes	Stable instrument performance

### Method comparison and practical implementation implications

The sorbent-coated metal disc method is compared with the conventional SPME method in Table 3, highlighting differences in key methodological aspects between the two methods.

**Table 3:** Method comparison table summarizing key aspects of the conventional SPME and sorbent-coated metal disc methods.

Method	SPME (conventional fiber)	Sorbent-coated metal discs
Sampling type	Snapshot, equilibrium-based	Time-integrated, capacity-driven
Extraction factor	Low–moderate; equilibrium-limited	High potential; mass- and time-dependent
LOD	Low (high sensitivity)	Moderate; qualitative/semi-quantitative
Time integration	No	Yes
Cost	Moderate (commercial fibers)	Low (bulk sorbent, reusable discs)
Complexity	Low	Moderate (multi-step workflow)

Table 3 shows that, although the current prototype stage of the sorbent-coated metal disc method results in moderate sensitivity and complexity, the novel method already shows potential for efficient extraction at a low cost. Based on the observations made in this work, several practical considerations relevant to method implementation can be identified, including the need to standardize sampling, the importance of monitoring background signals using blanks, and the need for consistent handling of the desorption and transfer steps. Taken together, these considerations indicate that the disc workflow can be applied reproducibly provided that key operational parameters are carefully controlled. The presented observations are intended to support implementation and interpretation of the workflow and should not be interpreted as a comprehensive analytical validation.

## Discussion

### Scope of the present work

The contribution of the present work is methodological. It formalizes sorbent disc sampling as a generalizable, platform-level architecture for time-integrated VOC enrichment prior to GC/MS analysis, specifies the protocols required for reproduction in other laboratories, and establishes the theoretical basis for interpreting signal formation in the capacity-driven, non-equilibrium regime in which the platform operates. The term "accuracy" in analytical chemistry has multiple legitimate definitions depending on the sampling and measurement regime, and the figure of merit appropriate to one regime is not necessarily appropriate to another. In the bioanalytical-method-validation sense codified by the ICH M10 guideline and the corresponding FDA Bioanalytical Method Validation Guidance for Industry [13], accuracy is defined as the closeness of agreement between a measured concentration and a known reference concentration, typically expressed as percent recovery against a calibrator of known nominal value. This definition presupposes a measurement regime in which the analyte is sampled exhaustively, or proportionately with a known partition coefficient, from a defined sample volume into the analytical system, so that the measured signal can be calibrated against a concentration in concentration units. Sorbent disc sampling, as formalized in the present manuscript, does not satisfy this presupposition. The disc operates under hybrid convective/diffusive transport in an open geometry with no defined volumetric sampling rate; the accumulated analyte mass is governed by the mass–transport competition framework S = M_ads × η_transfer rather than by a partition equilibrium with a defined sample volume. The platform-level signal therefore reflects time-integrated cumulative exposure, not concentration in the bioanalytical sense, and recovery against a reference concentration is not a meaningful figure of merit at the platform level. Equilibrium-based microextraction techniques face analogous, though not identical, definitional issues, and the SPME literature has long distinguished equilibrium SPME (where partition coefficients enable recovery calculation) from non-exhaustive pre-equilibrium SPME (where they do not) [14]. What is reported in the present manuscript instead are figures of merit appropriate to the platform regime. For specific applications of the platform that require concentration-based accuracy figures, the disc must be calibrated against a defined sampling geometry and a controlled sample volume. The clinical lung-cancer-screening application reported in the companion publication [2] addresses these requirements within the constraints of that application; analogous concentration-based validation in other application contexts is identified as future work.

### Position relative to existing sampling modes

The configurable architecture described in this work occupies a sampling regime that is distinct from the three established modes against which it is naturally compared: solid-phase microextraction (SPME), thin-film microextraction (TFME), and active thermal-desorption (TD) tube sampling. The distinctions are differences in sorbent mass, sorbent geometry, transport regime, and the relationship between sampling time and signal formation. They also have direct consequences for which compound classes the workflow can be expected to enrich efficiently and for the kind of validation that is appropriate to the platform.

SPME and TFME, as introduced and developed by Pawliszyn and co-workers, are equilibrium-based microextraction techniques. Their defining property is that analytes partition between the sample and a small sorbent phase, either a polymer-coated fiber in SPME or a continuous polymeric film on a thin support in TFME, until equilibrium is reached or approached. The absolute sorbent mass in both cases lies in the milligram range, and the sampling is non-exhaustive by design. The figure of merit that governs performance is the partition coefficient, and the central kinetic question is the time required to reach equilibrium. By contrast, the present work uses approximately 0.2 g of granular sorbent (Carbopack X, Tenax TA, or Porapak Q) immobilized on a planar metal substrate by silicone adhesive resulting in two orders of magnitude more sorbent mass than typical TFME loadings, and a particulate rather than continuous-film geometry. With a sampling duration of 60 min, the sorbent does not reach equilibrium with the surrounding gas phase; analyte uptake is governed instead by accumulation against a finite capacity, with signal increasing approximately with exposure time until the saturation regime is approached.

Active TD tube sampling occupies the opposite end of the comparison. TD tubes contain comparable or larger absolute masses of granular sorbent, but the sample is drawn through the tube at a defined volumetric flow rate using a pump or a controlled diffusion barrier, so that analyte uptake per unit time is constrained by the flow–volume relationship and a defined sampling rate can be calculated. The disc described here carries no such constraint. Its geometry is open, its transport regime is hybrid convective/diffusive (advected breath flow combined with diffusion across the boundary layer at the sorbent surface), and there is no defined volumetric sampling rate. In return for this loss of volumetric definability, the platform gains practical advantages: minimal patient or operator burden and the ability to be deployed in geometries that a tube format cannot match.

The mass–transport competition framework introduced in Fig. 2 formalizes the resulting position. The measurable signal is governed by S = M_ads × η_transfer, in which the accumulation term M_ads grows with sorbent mass and exposure time and the transfer term η_transfer falls for highly volatile analytes during thermal desorption and headspace transfer. Equilibrium-based microextraction does not face this trade-off, because the analyte is not accumulated to capacity in the first place; flow-controlled TD sampling does not face it in the same form, because the sampling-rate definition allows a direct conversion between accumulated mass and ambient concentration. Sorbent disc sampling sits between these regimes, and the framework specifies how its signal is formed and why the volatility-dependent behavior observed for analytes such as acetone is the expected consequence of the mechanism rather than a deficiency of the implementation.

GC/MS remains the reference method for structural identification of VOC mixtures. Alternatives to GC/MS for VOC analysis include direct-injection real-time methods such as proton-transfer-reaction mass spectrometry (PTR-MS) and selected-ion flow-tube mass spectrometry (SIFT-MS) [7,15,16], spectroscopic methods such as photoacoustic spectroscopy [17], chemical-sensor arrays and electronic noses [18], and biological-recognition approaches such as canine olfaction [19]. The use of GC/MS does carry recognized disadvantages, principally the energy and reagent intensity of the analytical platform itself (addressed in the Green-chemistry profile subsection) and the requirement for trained operators and laboratory infrastructure. However, none of the alternatives provide the structural identification confidence of GC/MS analysis.

### Method performance indicators

The performance indicators reported in this manuscript are meant to reflect the sorbent disc concept in general rather than the analytical performance of a single sorbent–substrate–instrument combination operating on a single matrix. For quantitative anchors expressed as enhancement factors, the companion publication [2] reports the disc workflow yielding GC/MS peak areas approximately 2–9 times larger than direct SPME for the four last-eluting standards in the seven-component mixture, and 5–166 times larger than Tedlar® bag + SPME for the eight semi-volatile compounds in the ten-compound breath panel. The measurements presented in Figs. 4 and 5 of the present manuscript are consistent in sign and approximate magnitude with these values.

### Matrix effects and contaminants

Matrix effects in VOC analysis of breath and ambient air arise from four principal sources: water vapor content of the sampled gas, CO₂ content (which can affect sorbent affinity), competitive adsorption of co-present semi-volatile compounds, and chemical or physical interaction of the sorbent surface with the matrix itself. Each of these is well-characterized in the breath-analysis and ambient-air-monitoring literature, and each is dependent on the sorbent chemistry [10,15,20].

Water-vapor is the most consequential matrix effect for breath-VOC sampling because exhaled breath is fully saturated at body temperature (∼44 g/m³ of water vapor at 37 °C) and condenses readily on cooler surfaces. On polar or oxidized sorbents, such as activated carbons with high surface oxygen content and ordered mesoporous carbons with hydrophilic functional groups, water adsorbs competitively with target VOCs and substantially reduces analyte recovery, particularly for less volatile analytes that depend on prolonged sorbent residence time [10]. Carbopack X, used here as the primary sorbent, is a graphitized carbon black with an essentially nonpolar sp² graphitic surface and is established in the literature as one of the more hydrophobic carbonaceous sorbents available [10,21]. The water-vapor effect on the present workflow is further attenuated by the headspace-transfer step, which presents the CAR/PDMS fiber with a small (∼15 mL) defined gas volume drawn from the desorption container, rather than with the entire breath sample; this geometric arrangement also reduces water co-elution onto the analytical column. Hartonen et al. [20] specifically address moisture effects in headspace SPME and document the conditions under which water co-extraction becomes analytically limiting; the present configuration operates within the conditions identified there as moisture-tolerant.

CO₂ effects on sorbent-based VOC sampling are typically negligible for carbonaceous sorbents at the partial pressures present in exhaled breath (∼5% CO₂), because CO₂ does not adsorb appreciably to graphitic surfaces at room temperature. CO₂ effects are not relevant for GC/MS analysis as opposed to direct-injection real-time methods such as PTR-MS [15]. Competitive adsorption among co-present VOCs is intrinsic to any capacity-limited sampling method, including the present platform. The mass–transport competition framework introduced in Fig. 2 accommodates competitive adsorption naturally as a contribution to the M_ads term: the accumulated mass of any individual analyte is constrained both by sorbent capacity and by the presence of competing adsorbates. Surface interaction effects are not expected for the sorbent–analyte combinations used here, because graphitized carbon blacks are chemically inert to the neutral organic VOCs targeted in breath analysis [10]. This is a particular advantage of graphitized carbons over more chemically active sorbents (some metal–organic frameworks, certain functionalized mesoporous carbons) where matrix-induced surface modification can alter sampling performance over time.

Other potential interferents include ambient-laboratory contaminants such as phthalates, GC column bleed, and septum-derived fragments [22]. These were monitored using procedural blanks throughout method development. Routine GC oven conditioning and the use of ultra-inert capillary columns minimize carry-through of these contaminants into the analytical window. The matrix-effect profile of the platform is therefore characterized by: limited water-vapor interference (from sorbent hydrophobicity and headspace-transfer geometry); negligible CO₂ interference (from the chromatographic separation step preceding MS detection); and no chemically active surface-modification effects. These are platform-level matrix-effect properties; specific applications of the platform may encounter additional matrix considerations depending on the sample type and the analyte panel.

### Memory effect and reusability

The memory-effect and reusability profile of the sorbent disc platform follows from the established behavior of graphitized carbon blacks in thermal-desorption-based VOC sampling. Carbopack-type sorbents are widely documented as reversible adsorbents that release accumulated analytes quantitatively at conditioning temperatures of 250–300 °C without irreversible binding, in contrast to microporous activated carbons where strong adsorbate–pore-wall interactions can lead to incomplete recovery and progressive deactivation across reuse cycles [10,21,23]. The mesoporous rather than microporous structure of Carbopack X is the structural basis for this reversibility: analytes do not become trapped in narrow micropores but instead reside on accessible mesopore and external surface sites that are vacated efficiently during thermal conditioning.

In the present work, conditioned discs were reused across multiple sampling cycles without observable carryover into subsequent analyses; the conditioned-disc blank chromatogram (Fig. 6) confirms the absence of residual analyte signal at target retention windows after the routine 250 °C / 30 min conditioning protocol. This is consistent with the behavior documented in the engineered-carbon-adsorbent literature for graphitized carbon blacks operated within their thermal-stability envelope. Disc lifetime in the present configuration is limited primarily by the mechanical integrity of the silicone-adhesive bond between the sorbent layer and the metal substrate after repeated thermal cycling, rather than by sorbent deactivation. Visual inspection of discs across the lifespan of the present study did not reveal sorbent-layer detachment or evident degradation.

### Mechanistic implications for users of the platform

The mass–transport competition framework S = M_ads × η_transfer, introduced in Fig. 2, has practical consequences for users of the platform that go beyond explanation of the volatility-dependent behavior observed in this work. It could also allow compound-specific behavior to be predicted. For analytes with boiling points above 100 °C the accumulation term M_ads dominates within the 60 min sampling window, and signal increases approximately linearly with sorbent mass and exposure time until saturation is approached. For analytes with boiling points below approximately 60 °C, exemplified by acetone in the present work, the transfer term η_transfer becomes limiting during the thermal-desorption and headspace-transfer step. The signal these analytes produce reflects what survives the two-step enrichment, not what was originally accumulated on the disc.

Several mitigations are available within the present design. Improved lid-to-cup sealing of the desorption container reduces η_transfer losses during heating; minimizing the delay between heating and headspace withdrawal limits diffusive escape from the desorption volume; controlling the re-adsorption temperature reduces re-volatilization of captured analytes from the SPME fiber; and reducing the evacuated headspace volume lessens dilution before re-adsorption. These mitigations operate within the existing two-step transfer architecture and do not require new components. More substantial improvements, for example incorporation of a Peltier-assisted cryo-focusing element into the desorption pathway to retain volatile analytes during transfer, represent future development directions rather than configuration changes within the present platform.

The framework also clarifies why the platform cannot be expected to reproduce the response profile of equilibrium-based microextraction or flow-controlled TD sampling for highly volatile analytes. The volatility dependence is a property of the regime, not of the implementation. The volatility-dependent signal behavior predicted by the mass–transport competition framework is grounded in established literature for graphitized carbon-black sorbents. Breakthrough volume is the threshold sample volume above which an analyte begins to elute from a sorbent bed during sampling, and it scales inversely with analyte volatility for any given sorbent at a given temperature [21,23]. For Carbopack X at room temperature, breakthrough volumes are tabulated in the manufacturer's product literature and in the analytical-chemistry literature for a range of common VOC analytes; values for highly volatile analytes such as acetone (boiling point 56 °C) and isoprene (boiling point 34 °C) are markedly lower than values for semi-volatile analytes such as decane (boiling point 174 °C) and benzaldehyde (boiling point 179 °C) [10].

In a sampling geometry such as the breath-jar configuration, the effective sampled volume is small relative to typical breakthrough thresholds for the sampling-stage temperatures and durations applied, and breakthrough during sampling itself is not the limiting process for the highly volatile analytes that show reduced platform response. Rather, the η_transfer term in the mechanistic framework captures the analogous loss process during the desorption-and-transfer step. At the 250 °C desorption temperature, highly volatile analytes are desorbed completely but escape partially during the 6 min heating window before the 15 mL headspace transfer to the SPME fiber. Highly volatile analytes also re-equilibrate less efficiently from the gas phase onto the CAR/PDMS fiber than semi-volatile analytes [9]. The two-step transfer architecture therefore inherits, in a different mechanistic form, the volatility scaling that breakthrough-volume considerations describe for direct-sampling thermal-desorption tubes. This continuity between established sorbent-tube behavior and the disc-platform behavior observed here strengthens the predictive use of the framework.

### Green-chemistry profile

**Fig. 7:**
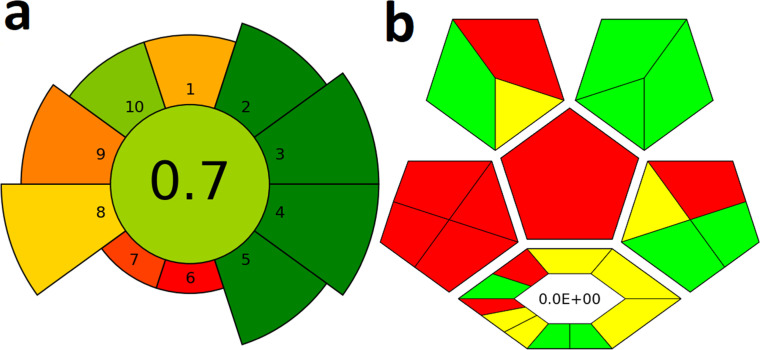
Green analytical assessment tool evaluation pictograms generated using corresponding freeware [24,25] for the sorbent disc workflow. (a) AGREEprep evaluation across the criteria 1: sample preparation placement (score = 0.33, weight = 3), 2: hazardous materials (score = 1.0, weight = 4), 3: sustainability, renewability, and reusability of materials (score = 1.0, weight = 5), 4: waste (score = 1.0, weight = 5), 5: size economy of the sample (score = 1.0, weight = 4), 6: sample throughput (score = 0.0, weight = 1), 7: integration and automation (score = 0.12, weight = 1), 8: energy consumption (score = 0.41, weight = 5), 9: post-sample preparation configuration for analysis (score = 0.25, weight = 4) and 10: operator's safety (score = 0.75, weight = 3) resulting in an overall score of 0.7. (b) ComplexGAPI evaluation across the pentagonal glyphs 1: method (middle), 2: sampling (left), 3: extraction (top left), 4: chemicals (top right) and 5: instrumentation (lower-right) as well as the hexagonal pre-analysis process glyph (bottom) including the numerical result of a waste generation quotient called the E-factor. The AGREEprep score is high since higher weights were given to the criteria that directly impact the environment and the sorbent disc workflow excel at all such criteria except criteria 8 and 9 due to the energy consumption of heating and GC/MS instrumentation. The AGREEprep score is also lowered by the method complexity which is more strongly emphasized and highlighted in the ComplexGAPI evaluation.

The workflow has been evaluated against two established green-analytical-chemistry assessment tools, AGREEprep [24] and ComplexGAPI [25], resulting in the pictograms in Fig. 7.

Several features of the platform contribute favorably to the assessment. The workflow is entirely solvent-free, and largely waste-free with reusable sorbent discs. Consumables are limited to small volumes of inert carrier gas, GC septa, and the silicone adhesive used at the disc preparation stage. The sampling step itself imposes no energy cost, and the conditioning and desorption steps use a standard GC oven and a small hot plate respectively. GC/MS is also energy- intensive, but remains the analytical platform required for structural identification of VOCs. The manual character of the disc preparation procedure limits the scalability of the workflow without contributing in itself to environmental burden. The cumulative material and energy footprint of the workflow is therefore low compared with sampling approaches that depend on disposable sorbent media or on solvent extraction. The full AGREEprep report is given in Table 4 and lists the scores and weights given to each criterion as well as the corresponding numerical and/or textual input data.

**Table 4:** Full AGREEprep table with detailed descriptions, inputs, scores, and weights for the ten evaluation criteria.

### Deployability and applicability across settings

A practical strength of the platform is that it places no infrastructure demands beyond those already met by standard analytical laboratories. Sampling requires no pump, no controlled volumetric flow, and no specialized field instrumentation; thermal desorption uses a sealed metal chamber and a laboratory oven; and analytical readout is performed on conventional split/splitless GC/MS systems of the kind installed in research and reference laboratories worldwide. The consumable cost per disc (< €5) and the reusability of the discs across multiple thermal cycles further reduce the per-sample burden relative to single-use sorbent tubes or pressurized canister sampling, both of which require either dedicated thermal-desorption interfaces or specialized handling and shipping. These features are consistent with deployment in resource-constrained settings, including occupational and indoor air-quality monitoring in low- and middle-income contexts [26], field sampling outside core laboratory infrastructure, and breath-based screening pilots where canister logistics would be prohibitive [27,28]. The disc format is also compatible with conventional postal shipping, simplifying multi-site collection in distributed studies. Field validation in such settings is beyond the scope of the present platform-level description, but the operational profile of the workflow makes these natural targets for follow-on application studies.

### Limitations

A limitation of the present platform configuration is the volatility-driven signal loss for analytes with boiling points below approximately 60 °C, exemplified by acetone in the present work. The mass–transport competition framework predicts this loss, the available mitigations within the existing design partially compensate for it, and the Peltier-assisted cryo-focusing approach indicated above offers a structural solution for future development. Within the present configuration the limitation should be acknowledged when designing studies on analyte panels in which highly volatile compounds carry diagnostic weight. Another limitation is the absence of a defined volumetric sampling rate. The disc operates under hybrid convective/diffusive transport conditions in an open geometry, without a controlled flow path. This means the platform produces relative-signal data, comparable across discs, sorbents, substrates, and instruments under standardized exposure conditions, rather than concentration measurements expressed per unit volume of sample. For applications in which absolute concentration values are required, the platform must be calibrated against a defined sampling geometry and a controlled volumetric flow, which is outside the scope of the present platform-level description. A third limitation is the manual preparation procedure that imposes a labor cost and a quality-control overhead that scale poorly. Automated application of the silicone adhesive and the sorbent powder, together with automated post-cure conditioning, would tighten the loading distribution and remove the manual labor bottleneck. Such automation would not change the platform’s mechanistic regime or its performance envelope; it would change only the production cost.

### Future perspectives

Specific applications of the platform require concentration-based analytical validation appropriate to the relevant matrix, regulatory context, and analyte panel; a clinical lung-cancer-screening application is already reported in the companion publication [2]. The configurable architecture demonstrated in Fig. 4 also opens the possibility of application-tailored sorbent selection from the broader catalogue of carbon-based and polymeric sorbents available, with the disc geometry itself preserved as a transferable platform element. A formal inter-laboratory transfer study would be useful for demonstrating the reproducibility of the platform.

## Conclusion

The present work establishes sorbent disc sampling as a platform-level architecture for time-integrated VOC enrichment prior to GC/MS analysis. The workflow consists of fabrication of a sorbent-coated metal disc by adhesive-mediated immobilization of a granular sorbent on a metal substrate, conditioning at controlled temperature and inert atmosphere, exposure of the conditioned disc to gaseous samples for 60 min, thermal desorption of the captured analytes, and transfer to a CAR/PDMS SPME fiber for GC/MS analysis. The disc architecture functions across exchangeable sorbents (Carbopack X, Tenax TA, Porapak Q) and across exchangeable metal substrates (a stainless-steel disc, a rectangular metal plate, a repurposed five-kronor coin), establishing the disc as a configurable architecture rather than a single device.

The conceptual contribution of the work is a mass–transport competition framework, S = M_ads × η_transfer, that describes signal formation in the capacity-driven, non-equilibrium regime in which the platform operates. The framework distinguishes sorbent disc sampling from equilibrium-based microextraction modes (SPME, TFME) and from flow-controlled active thermal-desorption tube sampling, predicts the volatility-dependent signal behavior observed for highly volatile analytes such as acetone. The framework also clarifies that the volatility-driven signal loss is a property of the sampling regime, not a deficiency of the implementation. The workflow is solvent-free, low-waste, and reusable, with a green-chemistry profile favorable on AGREEprep and ComplexGAPI metrics within the constraints imposed by the use of GC/MS as the determination instrument.

## Ethics statement

Not applicable, all breath samples were taken from the corresponding author.

## CRediT authorship contribution statement

**Robert Lundberg:** Formal analysis, Investigation, Validation, Visualization, Writing – original draft, Writing – review & editing. **Thomas Lundeberg:** Conceptualization, Funding acquisition, Writing – review & editing. **Johan Lundeberg:** Writing – review & editing. **Johan Dahlén:** Project administration, Supervision, Writing – review & editing.

## Declaration of competing interest

The authors declare that they have no known competing financial interests or personal relationships that could have appeared to influence the work reported in this paper.

## Data Availability

Data will be made available on request.
